# A Program‐Led Motivational App (MI‐Coach: ED) for Eating Disorder Waitlists: Findings From a Feasibility and Acceptability Pilot Trial

**DOI:** 10.1002/eat.70053

**Published:** 2026-02-06

**Authors:** Amané Halicki‐Asakawa, Emily Fuller, Maya Libben

**Affiliations:** ^1^ Department of Psychology University of British Columbia Kelowna British Columbia Canada

**Keywords:** acceptability, digital intervention, eating disorders, feasibility, mobile app, motivation, pilot study, pre‐treatment engagement, program‐led, waitlist

## Abstract

**Objective:**

Individuals with eating disorders (EDs) often face significant barriers to accessing care, including prolonged waitlists and systemic delays. Digital interventions, such as mobile apps, offer a scalable way to enhance pre‐treatment engagement during this high‐risk period. This pilot study evaluated the feasibility and acceptability of *MI‐Coach: ED*, a program‐led mobile app designed to support motivation among female‐identifying individuals awaiting ED treatment.

**Method:**

Twenty‐three female‐identifying individuals on waitlists at ED clinics across British Columbia, Canada, participated in a 4‐week single‐arm pilot trial of *MI‐Coach: ED*. The app delivered motivational interviewing–informed content through seven sequential modules containing reflective exercises, psychoeducational articles, and psychologist‐led videos. Feasibility was assessed via service‐provider uptake, participant enrollment, engagement, and retention metrics. Acceptability was measured using the user version of the Mobile App Rating Scale and adapted Technology Acceptance Model ratings. Exploratory analyses descriptively examined pre‐to‐post changes in motivation and related symptoms.

**Results:**

Feasibility was constrained at the service‐provider level, with 6% of contacted sites agreeing to distribute study materials. At the participant level, 67.6% initiated app use and 44% completed at least four modules, and 78% completed pre‐ and post‐assessments, indicating partial engagement across the sample. Participants rated the app positively across domains of quality, ease of use, and perceived usefulness. Small‐to‐medium reductions in global ED severity and depressive symptoms, as well as increases in motivational confidence, were observed with confidence intervals that did not cross zero. Change scores were moderately correlated across select motivational and symptom measures.

**Discussion:**

Findings suggest that *MI‐Coach: ED* was acceptable among users who engaged, while feasibility was substantially influenced by system‐level recruitment constraints and variable participant engagement. Observed engagement patterns were lower than those reported for general mental health apps but consistent with prior digital ED intervention literature, underscoring the importance of disorder‐specific feasibility benchmarks. Results will inform ongoing refinements and implementation strategies for a future randomized trial.

## Key Points

- Eating disorder treatment often involves long wait times before accessing care.
- This study evaluated a motivational mobile app designed to support individuals during this critical waiting period.
- Findings suggest that digital, program‐led tools like *MI‐Coach: ED* may offer accessible support to enhance motivation and treatment readiness.

## 1 Introduction

Although eating disorders (EDs) are associated with high mortality and significant medical risk, access to timely care remains limited. Delays between symptom onset and treatment often span years (Austin et al. 2021), and individuals with EDs are significantly less likely to access mental health services compared to other clinical populations (Innes et al. 2017; Weissman et al. 2020). These prolonged wait periods carry serious clinical implications: longer wait times predict dropout (Carter et al. 2012), early attrition from services (Blackburn et al. 2024), and, in the case of anorexia nervosa, increased mortality risk (Solmi et al. 2024). Qualitative accounts describe the toll of passive wait periods, with worsening symptoms and reduced motivation (Goode et al. 2023; Treasure et al. 2024). Furthermore, extended delays are associated with neurobiological adaptation and the reinforcement of maladaptive behavioral patterns, which may further undermine treatment outcomes (O'Hara et al. 2015; Steinglass and Walsh 2016).

In light of these findings, there is growing interest in improving engagement during waitlist periods (Wade et al. 2024). However, multiple barriers undermine early engagement in treatment, including shame, ambivalence, and internalized stigma (Ali et al. 2020; Lubieniecki et al. 2024), as well as clinician burnout, limited availability, and rigid intake protocols (Innes et al. 2017). Many patients either decline or disengage from care altogether, citing poor fit, emotional unreadiness, and inflexible programming (Andersen et al. 2021; Liu et al. 2022; Sheridan and McArdle 2016; Vinchenzo et al. 2021). These findings underscore the need for structured, flexible, and accessible pre‐treatment supports that address both systemic and individual barriers.

Program‐led interventions offer a promising solution by embedding focused, structured support between referral and formal treatment. Defined as interventions delivered outside of traditional therapy, often with minimal clinician involvement, they can be implemented digitally or through self‐guided formats to reduce clinician workload and expand access (Davey et al. 2023; LaMarre et al. 2025; Wasil et al. 2021). One modifiable target that may be well‐suited to this intervention format is motivation. Originally developed for substance use disorders, motivational interviewing (MI) is a client‐centered approach that aims to resolve ambivalence by exploring the discrepancy between an individual's values and/or goals, and their current behaviors (Weiss et al. 2013). MI has been adapted for use in a range of formats within ED care, including peer support delivery, guided self‐help, and group‐based contexts, and has demonstrated efficacy as a pre‐treatment tool (Feld et al. 2001; Weiss et al. 2013). Higher pre‐treatment motivation is associated with stronger treatment engagement and lower relapse risk (Cruz et al. 2025), making it a compelling intervention target during wait periods.

Mobile apps may offer a scalable delivery vehicle for delivering program‐led, MI‐based interventions. App‐based tools have demonstrated preliminary efficacy in reducing ED symptoms (Cruz et al. 2025) and promoting broader health behavior changes (Chandrashekar 2018; Han and Lee 2018; Lee et al. 2018). Furthermore, a systematic review by Wasil et al. (2021) found that ED intervention apps with motivational features were more widely used, suggesting that users actively seek tools that enhance readiness for treatment. However, many existing ED apps lack evidence‐based content and fail to leverage the capabilities of mobile platforms for engagement and personalization (Juarascio et al. 2015; Kim et al. 2018; Sadeh‐Sharvit et al. 2018; Tregarthen et al. 2019). For example, a qualitative evaluation by Lindgreen et al. (2018) found that ED app users value structure and guidance, and disengage when content feels generic, impersonal, or disconnected from lived experience. Taken together, these findings highlight an opportunity to develop MI‐informed apps that are sufficiently structured to guide users while remaining flexible to individual motivation trajectories.

Given the lack of structured, scalable supports during ED treatment waitlists, a program‐led mobile app grounded in MI was developed to address this gap. *MI‐Coach: ED* was adapted from an existing digital platform (*MI‐Coach*, Resiliens Inc.) originally designed to support motivation for health behavior change (e.g., physical activity, smoking cessation, sleep hygiene). Consistent with best practices for digital ED interventions (Linardon et al. 2022), the app was tailored to meet the unique motivational needs of individuals with EDs. Full details regarding the development process are reported elsewhere (Halicki‐Asakawa, Mocci, and Libben 2025). The final app includes seven sequential, self‐guided MI‐based modules designed to support readiness for recovery during the waitlist period.

A mixed‐methods pilot evaluation of *MI‐Coach: ED* was conducted with women on ED waitlists to examine how the app functioned in routine care, how users engaged with its weekly content, and whether it offered meaningful support during the pre‐treatment period. To deepen understanding of lived experiences and to ensure that development remained grounded in participatory design principles, the pilot incorporated a framework matrix analysis of semi‐structured interviews conducted with interest‐holder groups (i.e., individuals with lived ED experience who participated in the pilot study, and clinicians working within ED treatment settings). Participants described *MI‐Coach: ED* as accessible and clinically relevant, noted clearer reasons for change and more manageable short‐term goals, and highlighted how the app could fit within existing care pathways. Additional details regarding the qualitative components of the pilot test are available in a separate publication (Halicki‐Asakawa, Gerlof, et al. 2025).

This article reports on pilot outcomes related to feasibility (i.e., recruitment, retention, and engagement rates), acceptability (i.e., ease‐of‐use, perceived usefulness), and exploratory indicators of potential clinical utility. Specifically, the study examined whether *MI‐Coach: ED* is feasible to implement in a real‐world waitlist context, acceptable to users, and associated with descriptive patterns of change in motivation and related symptoms over 4 weeks. This feasibility‐focused pilot was not designed to evaluate efficacy, but rather to inform the development of a future randomized controlled trial (RCT) and the design of scalable, program‐led interventions for ED waitlist populations.

## 2 Methods

### 2.1 Design

This study used an uncontrolled, single‐arm pilot feasibility trial with no allocation, conducted over a 4‐week period. Reporting follows the CONSORT extension for pilot trials (Eldridge et al. 2016), which is provided inSupporting Information. This pilot was not prospectively registered, as it was designed to assess feasibility and inform the development of a subsequent definitive trial. No changes were made to pilot trial assessments or procedures after the trial commenced. The study received ethical approvals from the University of British Columbia Behavioral Research Ethics Board (H22‐02046) and the Vancouver Coastal Health Research Institute Ethics Board (V22‐02046). The larger randomized controlled trial, which is currently underway, is registered with ClinicalTrials.gov (NCT06801197).

### 2.2 Intervention

*MI‐Coach: ED* is a seven‐module, program‐led mobile intervention grounded in MI principles and delivered sequentially over 4 weeks. Module content targets core MI processes, including engagement, self‐compassion, focus‐setting, ambivalence and change talk, commitment and planning, and relapse prevention. Each module combines brief psychoeducational content and asynchronous clinician‐led videos with structured interactive exercises, such as guided self‐reflection, values clarification, ambivalence exploration, confidence and importance scaling, and change planning. The program includes 34 interactive exercises across modules, designed to support reflection and readiness for change rather than symptom monitoring. Detailed session‐level content is reported in the published protocol (Halicki‐Asakawa, Gerlof, et al. 2025).

### 2.3 Participants

#### 2.3.1 Eligibility

Participants were female‐identifying adults (≥ 18 years) recruited from ED treatment waitlists across British Columbia (BC), Canada. The sample was limited to women to reduce heterogeneity in motivational processes in this early‐stage pilot and to reflect the predominantly female composition of ED waitlists (Forrest et al. 2017; Hart et al. 2011). Eligibility criteria included: (1) placement on an ED‐specific treatment waitlist in BC; (2) age 18 years or older; (3) self‐identifying as a woman; (4) fluency in English; (5) self‐reported current or past diagnosis of a threshold or subthreshold ED (BN, AN‐R, AN‐BP, BED, OSFED); (6) no history of psychosis or schizophrenia‐spectrum disorder; and (7) no cognitive or sensory impairments that would interfere with app use (e.g., hearing impairments, recent traumatic brain injury).

### 2.4 Recruitment and Procedure

Participants were recruited between November 2022 and May 2024. Clinicians and administrators at provincial health authorities, non‐profit charities, and private practice clinics disseminated recruitment flyers. Recruitment also occurred through online platforms (e.g., social media, ED information websites). Interested participants contacted the research team via email, after which a15‐minute telephone screener was conducted by the study team to assess eligibility and to provide additional details regarding the study procedure. As part of the screening procedure, the Sick, Control, One, Fat, and Food ([SCOFF]; Morgan et al. 1999) questionnaire, a brief screening tool used to evaluate eating pathology, was administered over the phone to screen for the presence of ED symptoms.

Once eligibility was confirmed, participants provided digital consent and completed baseline questionnaires via Qualtrics, with diagnostic status confirmed through self‐reported ED diagnosis, age of diagnosis, and illness duration. Participants were then enrolled and attended a virtual onboarding session where they were guided through app installation and instructed to begin the program immediately after the onboarding session. Participants were encouraged to complete one to two sequential modules per week over 4 weeks and otherwise used the app independently. Full details regarding the app development process, content framework, and delivery format are detailed in the published pilot study protocol (Halicki‐Asakawa, Mocci, and Libben 2025). Following program completion, participants were invited to a qualitative interview reported separately (Halicki‐Asakawa, Gerlof, et al. 2025). Participants received a $40 CAD gift card upon completion. Enrollment proceeded on a rolling basis. Recruitment occurred on a rolling basis, and the trial concluded once the target sample size was reached.

### 2.5 Outcome Measures

#### 2.5.1 Feasibility Metrics

Primary feasibility outcomes were recruitment rate (percentage of contacted participants enrolled), retention (percentage completing post‐test assessments), and participant engagement (app logins, duration of app use, module/exercise completion rates). App usage metrics were collected through a clinician dashboard, which allowed the study team to access and monitor participants' app use throughout the course of the study.

#### 2.5.2 Acceptability

App acceptability was assessed using the User Version of the Mobile App Rating Scale ([uMARS] Stoyanov et al. 2016), evaluating engagement, functionality, aesthetics, information quality, and subjective app quality. The Technology Acceptance Model ([TAM]; Davis 1989) questionnaire measured perceived ease of use and usefulness of the app.

#### 2.5.3 Clinical Characteristics

Participant baseline sociodemographic characteristics (e.g., gender identity, age, education level, ethnicity) and medical history (e.g., history of ED treatment, age of ED onset, comorbid psychiatric conditions) were assessed using a self‐report questionnaire. Baseline technological literacy was assessed via the eHealth Literacy Scale ([eHEALS]; Norman and Skinner 2006) and the Mobile Device Proficiency Questionnaire ([MDPQ]; Roque and Boot 2018). Clinical measures evaluated eating pathology (Eating Disorder Examination Questionnaire [EDE‐Q]; Fairburn and Beglin 1994), motivation (Readiness and Motivation Questionnaire [RMQ]; Geller et al. 2013), body dissatisfaction (Body Shape Questionnaire [BSQ]; Cooper et al. 1987), depression (Patient Health Questionnaire [PHQ‐9]; Kroenke et al. 2001), and anxiety (General Anxiety Disorder‐7 [GAD‐7]; Spitzer et al. 2006). Each measure was selected for its relevance to ED symptomatology and related mental health symptoms and supported by published reliability and validity data. No changes were made to pilot trial assessments or procedures after the trial commenced.

### 2.6 Analytic Approach

Analyses were performed using R (version 4.2.0). Participants who did not complete post‐intervention assessments and had not initiated use of the app were excluded from pre‐post clinical change analyses using listwise deletion. Demographic characteristics, feasibility metrics, and acceptability ratings were summarized using means, standard deviations, and frequencies.

To characterize preliminary change over time, individual change scores (post‐pre) for each outcome (i.e., depressive symptoms, anxiety symptoms, global eating‐disorder severity, body dissatisfaction, and each readiness‐to‐change subscale) were computed. Given the pilot nature of the study and limited sample size, analyses were explicitly descriptive and estimation focused. Effect sizes for within‐person change were summarized using Cohen's d (standardized mean change) with 95% confidence intervals (CI), rather than relying on null hypothesis significance testing. No formal hypothesis tests were used to evaluate pre–post differences. The interrelationships among these change scores were subsequently examined by calculating Pearson correlations to examine patterns of co‐occurrence across motivational and symptom measures. Correlation analyses were conducted for exploratory purposes only and visualized using a clustered heatmap.

Clinical significance was additionally examined using the Reliable Change Index (RCI; Jacobson and Truax 1992) to descriptively classify individual‐level change as *recovered, non‐reliably recovered, improved, unchanged*, or *deteriorated* based on measure‐specific reliability estimates and clinical cut‐offs (Jacobson and Truax 1992; Lambert and Bailey 2012). Given the small sample size, these classifications were interpreted cautiously as descriptive indicators of variability rather than as estimates of population‐level effects.

A priori criteria for progressing to a full trial included: (1) ≥ 50% enrollment, (2) ≥ 70% retention, and (3) positive app usability ratings (mean score > 3.5 on the uMARS). These benchmarks were used pragmatically to guide study planning rather than as strict thresholds, consistent with recommendations for pilot and usability research (Eldridge et al. 2016; Lewis 2014; Thabane et al. 2010). As a feasibility‐focused pilot not powered to assess efficacy, no formal sample size calculation was conducted. Instead, a pragmatic target of approximately 20–25 participants was selected to support descriptive estimation of feasibility and acceptability, in line with guidance emphasizing flexibility in pilot sample sizes (Eldridge et al. 2016; Julious 2005). No interim analyses or stopping guidelines were employed.

## 3 Results

### 3.1 Feasibility Outcomes

#### 3.1.1 Study Recruitment and Site Participation

A total of 131 ED treatment centers were identified across BC. However, contact information was only available for 68% of sites. Of those that could be reached, response rates were low. Only 6% of contacted sites (*n* = 8) agreed to disseminate study materials to their clients. Participating sites included public treatment centers, advocacy organizations, private practices, and individual clinicians. To support recruitment, the study also employed community‐based outreach, including online advertisements (e.g., National Eating Disorder Information Centre) and social media posts (e.g., Twitter).

**FIGURE 1:**
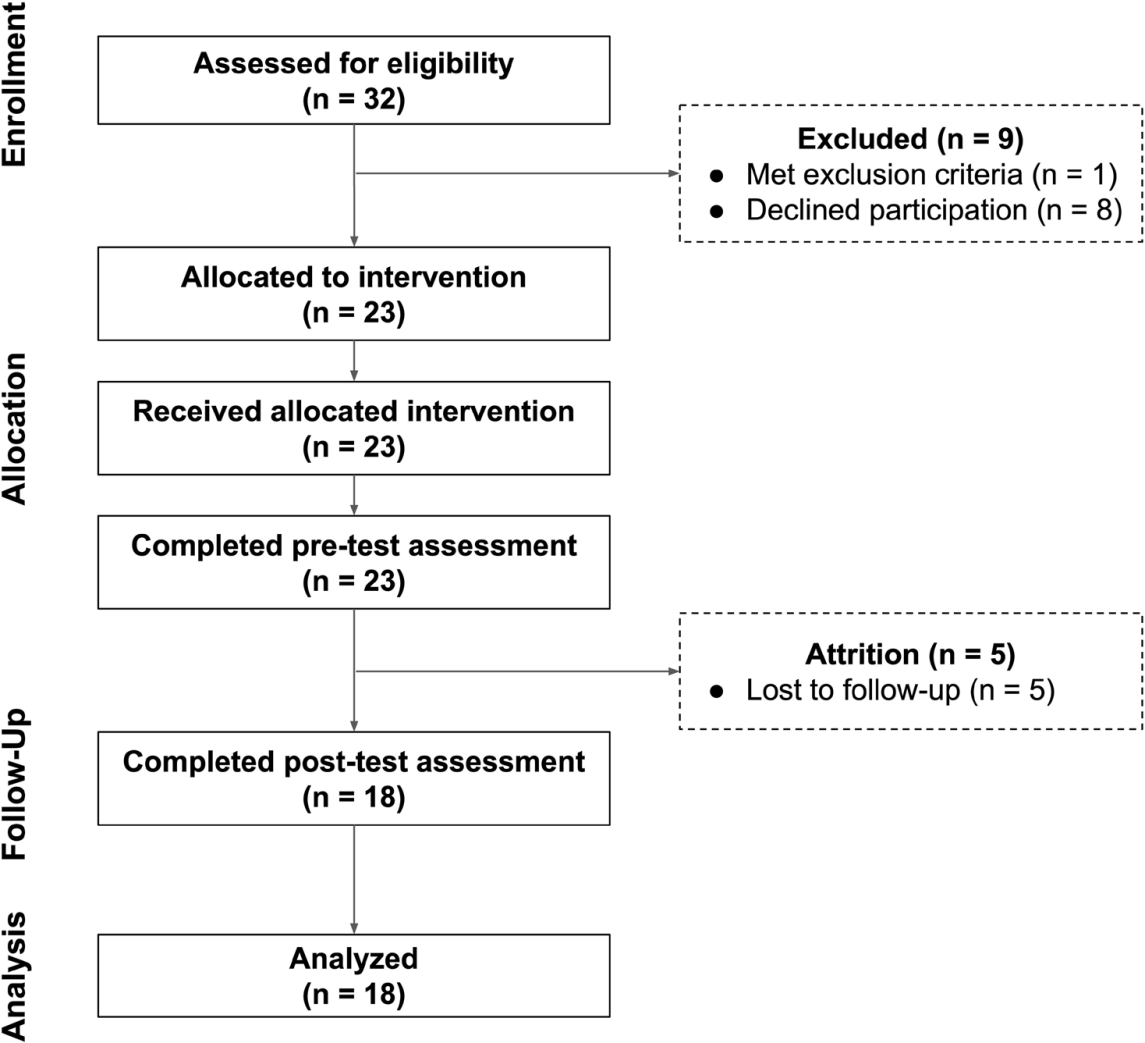
CONSORT study flow diagram. Flow of participants through the feasibility trial in accordance with the CONSORT recommendations for pilot and feasibility studies.

Of the 34 individuals initially assessed, one was excluded due to ineligibility, and eight declined participation following screening, yielding an enrollment rate of 67.6% (*N* = 23). The final sample included 23 female‐identifying individuals aged 22–65 (*M* = 35.1, SD = 11.4), who self‐identified as having an ED and were actively waitlisted for treatment. Of these, five participants were lost to follow‐up, resulting in an attrition rate of 21.7% (see Figure 1 for flow diagram). All participants lost to follow‐up did not initiate use of the app and were therefore excluded from pre‐post clinical change analyses.

### 3.2 Baseline Characteristics

Participants reported high comfort and proficiency with mobile technology, supported by strong scores on the eHEALS (*M* = 3.47, SD = 0.63) and MDPQ (*M* = 38.89, SD = 1.46), indicating high feasibility for digital delivery. On average, participants reported the onset of ED symptoms in adolescence (*M* = 16.22 years, SD = 7.25) and had been on a treatment waitlist for over 8 months (*M* = 34.32 weeks, SD = 48.47), reflecting enduring illness and delayed service access, as well as substantial variability in wait times across the sample. Clinically, participants endorsed elevated symptoms on the EDE‐Q Global score (*M* = 3.93, SD = 1.46) and substantial body dissatisfaction on the BSQ (*M* = 127.68, SD = 43.11). Moderate depressive and anxiety symptoms were observed on the PHQ‐9 (*M* = 14.05, SD = 6.03) and GAD‐7 (*M* = 11.82, SD = 5.97), respectively. Motivation ratings reflected moderate ambivalence on the RMQ Precontemplation subscale (*M* = 69.42, SD = 21.54), suggesting that many participants felt unready to change. Action (*M* = 37.97, SD = 24.20) and Internality (*M* = 59.98, SD = 25.61) scores fell in the mid‐to‐upper range, indicating some recognition of the need for change and feelings of ownership over recovery. In contrast, Confidence scores were on the lower end (*M* = 29.27, SD = 15.57), suggesting lower perceived ability to make changes. See Table 1 for full demographic characteristics and Table 2 for baseline clinical characteristics and technological literacy.

**TABLE 1:** Demographic characteristics (*N* = 23).

Variable	*n* (%)
Comorbid diagnoses^a^	
Psychiatric condition	15 (65)
Physical health condition	8 (35)
Ethnicity^a^	
White/Caucasian	22 (96)
South Asian	1 (4.3)
Highest degree earned	
High school	3 (13)
Some university/college credit, no degree	8 (35)
Bachelor's degree	8 (35)
Graduate degree	4 (17)
Average yearly income^b^	
<$29,999	6 (26)
$30,000–$49,999	4 (17)
$50,000–$69,999	3 (13)
$70,000–$89,999	4 (17)
$100,000–$149,999	3 (13)
Other	1 (4.3)
Prefer not to answer	1 (4.3)

*Note*: Percentages are based on a total sample size of *N* = 23. Categories are mutually exclusive unless otherwise noted. Variable labels reflect self‐reported demographic information. Education and income categories were collapsed for clarity.

^a^ Categories are not mutually exclusive.

^b^ Income reported in Canadian dollars (CAD).

**TABLE 2:** Baseline clinical characteristics and technological literacy (*N* = 23).

Variable	*M* (SD)
Clinical characteristics	
Age (years)	35.09 (11.42)
BMI	23.80 (6.06)
Age of ED onset (years)	16.22 (7.25)
Duration of ED (years)	18.87 (12.00)
Time spent on treatment waitlist (weeks)	34.32 (48.47)
RMQ	
Precontemplation	69.42 (21.54)
Action	37.97 (24.20)
Internality	59.98 (25.61)
Confidence	29.27 (15.57)
EDE‐Q (Global)	3.93 (1.46)
PHQ‐9	14.05 (6.03)
GAD‐7	11.82 (5.97)
BSQ	127.68 (43.11)
Technological literacy	
eHEALS	3.47 (0.63)
MDPQ	38.89 (1.46)

Abbreviations: BMI = body mass index; BSQ = body shape Questionnaire; ED = eating disorder; EDE‐Q = eating disorder examination questionnaire; eHEALS = eHealth literacy scale; GAD‐7 = generalized anxiety disorder scale–7; *M* = mean; MDPQ = mobile device proficiency questionnaire; PHQ‐9 = patient health questionnaire–9; RMQ = readiness and motivation questionnaire; SD = standard deviation.

### 3.3 Engagement, Acceptability, and User Experience

Participants used the app for a median of 16.5 days (range = 1.00–35.00) across the 4‐week pilot study period. Approximately 44% of participants completed more than half of the seven available sessions (≥ 4 modules), with one participant (5.6%) completing all seven modules, and the median number of sessions completed was 4.5. Engagement with interactive exercises was more variable, as while the median number completed was 8.5, some participants engaged with the app extensively, completing up to 34 exercises across modules. Medians are reported given the skewed distribution of app use variables and greater interpretability for small samples.

**FIGURE 2:**
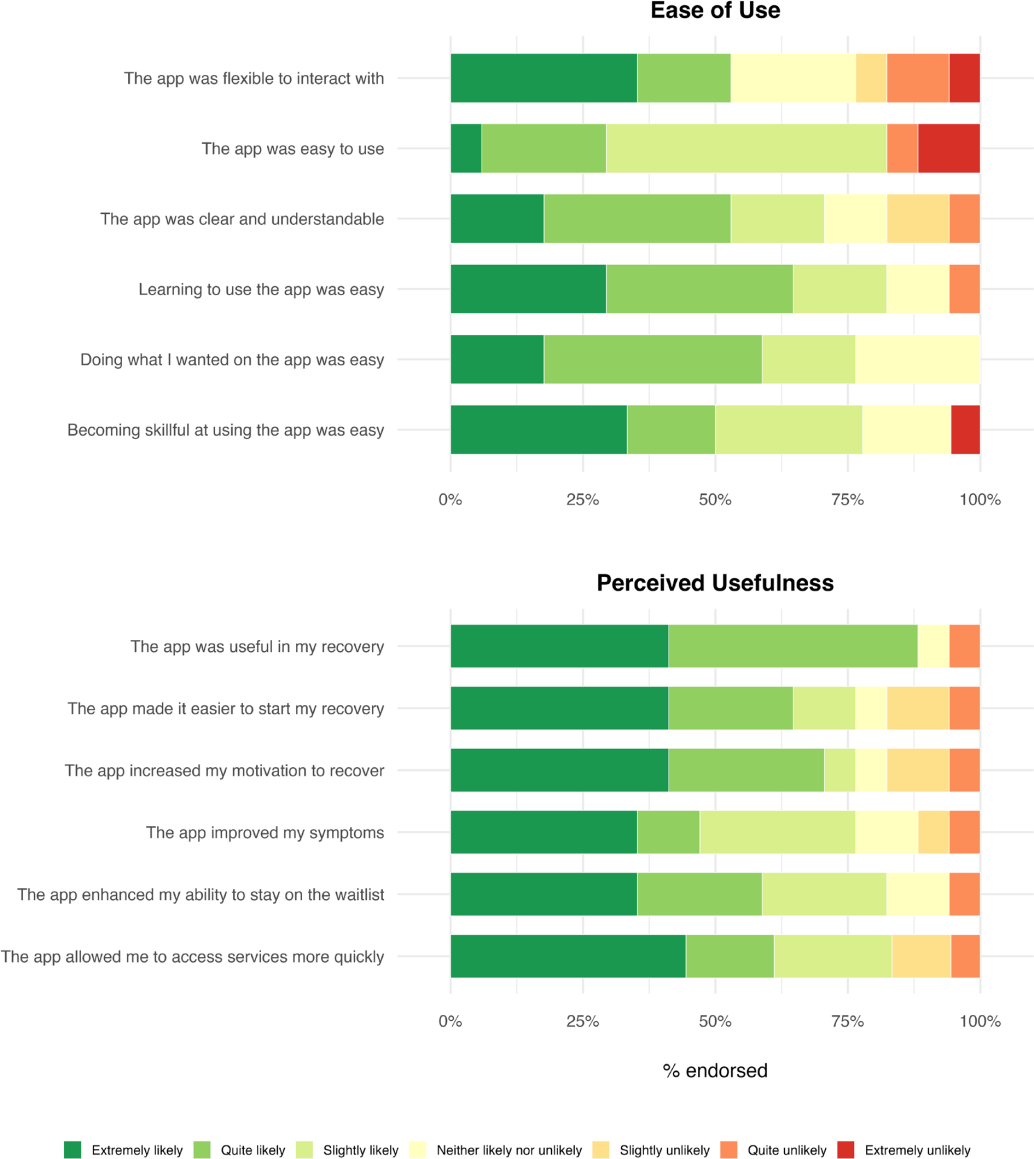
Participant ratings of app ease of use and perceived usefulness (*n* = 18). Stacked bar plots display the proportion of participants endorsing each response category across two domains: Ease of use (top panel) and perceived usefulness (bottom panel). Items were rated on a 7‐point Likert scale ranging from *extremely likely* (dark green) to *extremely unlikely* (red). Higher proportions of green reflect more favorable user ratings.

In contrast, app quality ratings were summarized using means and standard deviations, which are appropriate for Likert‐type response formats. On the uMARS, participants described the app as functional (*M* = 4.03, SD = 0.87), aesthetically pleasing (*M* = 4.20, SD = 0.55), informative (*M* = 4.24, SD = 0.88), and moderately engaging (*M* = 3.43, SD = 0.75). Overall app quality was highly (*M* = 3.98, SD = 0.54). On the adapted TAM, participants endorsed high ease of use (*M* = 5.73, SD = 1.41) and usefulness (*M* = 5.19, SD = 1.19). See Table 3 for full descriptive statistics and Figure 2 for proportions of participants endorsing high app satisfaction. No adverse events, safety concerns, or unintended effects were reported.

**TABLE 3:** App usage and user experience ratings (*n* = 18).

Variable	*M* (SD)	Median	Range
App usage			
Duration of consistent use (days)	16.58 (8.93)	16.50	1.00–35.00
Frequency of logins (days)	4.64 (3.20)	4.00	1.00–11.00
Sessions completed^a^	4.00 (2.15)	4.50	1.00–7.00
Exercises Completed^b^	10.50 (8.65)	8.50	3.00–34.00
User experience ratings			
uMARS scores			
Engagement	3.43 (0.75)	3.40	1.80–4.95
Functionality	4.03 (0.87)	4.25	1.75–5.00
Aesthetics	4.20 (0.55)	4.17	3.33–5.00
Information	4.24 (0.88)	4.38	1.25–5.00
Overall app quality	3.98 (0.54)	3.92	2.68–4.80
Adapted TAM scores			
Ease of use	5.73 (1.41)	6.33	2.00–7.00
Usefulness	5.19 (1.19)	5.67	2.67–6.67

*Note*: Higher scores indicate more favorable app evaluations.

Abbreviations: *M* = mean; SD = standard deviation; TAM = technology acceptance model (adapted version; range = 1–7); uMARS = user version of the mobile app rating scale (range = 1–5).

^a^ Maximum possible sessions = 7.

^b^ Number of completed interactive exercises across all modules.

### 3.4 Descriptive Patterns of Clinical Change

Table 4 presents the reliable change data across all RMQ subscales. On the Precontemplation subscale, 17 participants were categorized as *unchanged*, and no participants were in the *improved* or *deteriorated* categories. On the Action subscale, one participant met statistical criteria for the *improved* category, while two participants were categorized as *deteriorated*. For the Internality subscale, one participant fell in the *improved category*, and one participant was categorized as *deteriorated*. Similarly, on the Confidence subscale, one participant fell in the *improved category*, and one was classified as *non‐reliably recovered*.

**TABLE 4:** Jacobson‐Truax clinical significance classifications across outcome measures.

Outcome variable	Deteriorated *n* (%)	Unchanged *n* (%)	Improved *n* (%)	Non‐reliably recovered *n* (%)	Recovered *n* (%)
RMQ					
	0 (0)	17 (100)	0 (0)	0 (0)	0 (0)
Action	2 (11.8)	14 (82.4)	1 (5.9)	0 (0)	0 (0)
Internality	1 (5.9)	15 (88.2)	1 (5.9)	0 (0)	0 (0)
Confidence	0 (0)	15 (88.2)	1 (5.9)	1 (5.9)	0 (0)
EDE‐Q (Global)	0 (0%)	11 (61.1)	5 (27.8)	2 (11.1)	0 (0)
PHQ‐9	1 (5.9)	13 (76.5)	1 (5.9)	2 (11.8)	0 (0)
GAD‐7	1 (5.6)	12 (66.7)	2 (11.1)	3 (16.7)	0 (0)
BSQ	0 (0%)	12 (66.7)	2 (11.1)	3 (16.7)	1 (5.6)

*Note*: Table presents the number and percentage of participants falling into each Jacobson‐Truax clinical significance category as descriptive indicators of variability in pre‐ to post‐assessment scores, and are not intended to indicate intervention effects or clinical efficacy. Analyses were conducted on a completer sample; within this sample, Jacobson–Truax classifications were calculated using available pre‐ and post‐data for each measure, resulting in varying sample sizes across outcomes. *Deteriorated* = condition worsened reliably; *Unchanged* = no reliable change; *Improved* = reliable improvement, but post‐score remained in the clinical range; *Non‐reliably Recovered* = post‐score in normative range but change not statistically reliable; *Recovered* = reliable and clinically significant change. Categories were calculated using measure‐specific reliability coefficients and cutoffs.

Abbreviations: BSQ = body shape questionnaire; EDE‐Q = eating disorder examination questionnaire; GAD‐7 = generalized anxiety DISORDER scale–7; PHQ‐9 = patient health questionnaire–9; RMQ = readiness and motivation questionnaire.

Exploratory analyses of symptom‐related outcomes documented variability in reliable change classifications. On the EDE‐Q Global score, five participants were classified as *improved*, and two as *non‐reliably improved*, with no participants falling in the *deteriorated* category. For the BSQ, one participant was classified as *improved* on self‐reported ratings, two were classified as *non‐reliably improved*, and one participant was classified as *deteriorated* with respect to self‐reported ratings. On the GAD‐7, two participants were classified as *improved*, three as *non‐reliably improved*, and one participant was classified as *deteriorated* with respect to self‐reported ratings. Lastly, for the PHQ‐9, two participants were classified in the *improved* category on self‐reported ratings, three as *non‐reliably improved*, and one participant was classified as *recovered*, with no participants categorized as *deteriorated*.

Descriptive change statistics and standardized effect size estimates (Cohen's dz) with corresponding 95% confidence intervals for both mean change and effect size are summarized in Table 5. For RMQ Confidence, EDE‐Q Global, and PHQ‐9, confidence intervals for both mean change and effect size did not include zero, indicating more stable estimates of change. In contrast, confidence intervals for most other outcomes included zero, indicating that observed changes were small, variable, and imprecisely estimated in this sample. Taken together, these results suggest that while the majority of participants fell within the *unchanged* category over the course of the pilot evaluation, a subset were classified into clinical significance categories (i.e., *improved, non‐reliably recovered, recovered*), with some participants also classified as *deteriorated*. Consistent with the feasibility‐focused and exploratory nature of the study, these classifications are reported to characterize variability in change during the waitlist period rather than to indicate intervention efficacy.

**TABLE 5:** Descriptive statistics and estimation‐based effect sizes for pre‐ to post‐intervention change (*n* = 18).

Outcome variable	Mean change		Effect size	
	Δ*M* (SD)	95% CI (Δ*M*)	Cohen's dz	95% CI (dz)
RMQ				
Precontemplation	−6.13 (17.13)	[−14.94, 2.68]	−0.36	[−0.85, 0.13]
Action	1.31 (24.46)	[−11.27, 13.89]	0.05	[−0.43, 0.53]
Internality	−1.83 (27.86)	[−16.16, 12.49]	−0.07	[−0.55, 0.41]
Confidence	5.97 (10.99)	[0.32, 11.62]	0.54	[0.03, 1.05]
EDE‐Q (Global)	−0.40 (0.62)	[−0.72, −0.08]	−0.65	[−1.17, −0.13]
PHQ‐9	−3.35 (4.73)	[−5.78, −0.92]	−0.71	[−1.24, −0.18]
GAD‐7	−1.00 (4.82)	[−3.48, 1.48]	−0.21	[−0.69, 0.27]
BSQ	−7.12 (18.46)	[−16.61, 2.37]	−0.39	[−0.88, 0.1]

Abbreviations: 95% CI (Δ*M*) = confidence interval for the mean change; BSQ = body shape questionnaire; Cohen's dz. = standardized mean change for paired data (Δ*M*/SDΔ); 95% CI (dz) = confidence interval for Cohen's dz: EDE‐Q = eating disorder examination questionnaire; GAD‐7 = generalized anxiety disorder–7; PHQ‐9 = patient health questionnaire–9; RMQ = readiness and motivation questionnaire; SD = standard deviation of the change score; Δ*M* = mean change score (post–pre).

### 3.5 Associations Among Change Scores

**FIGURE 3:**
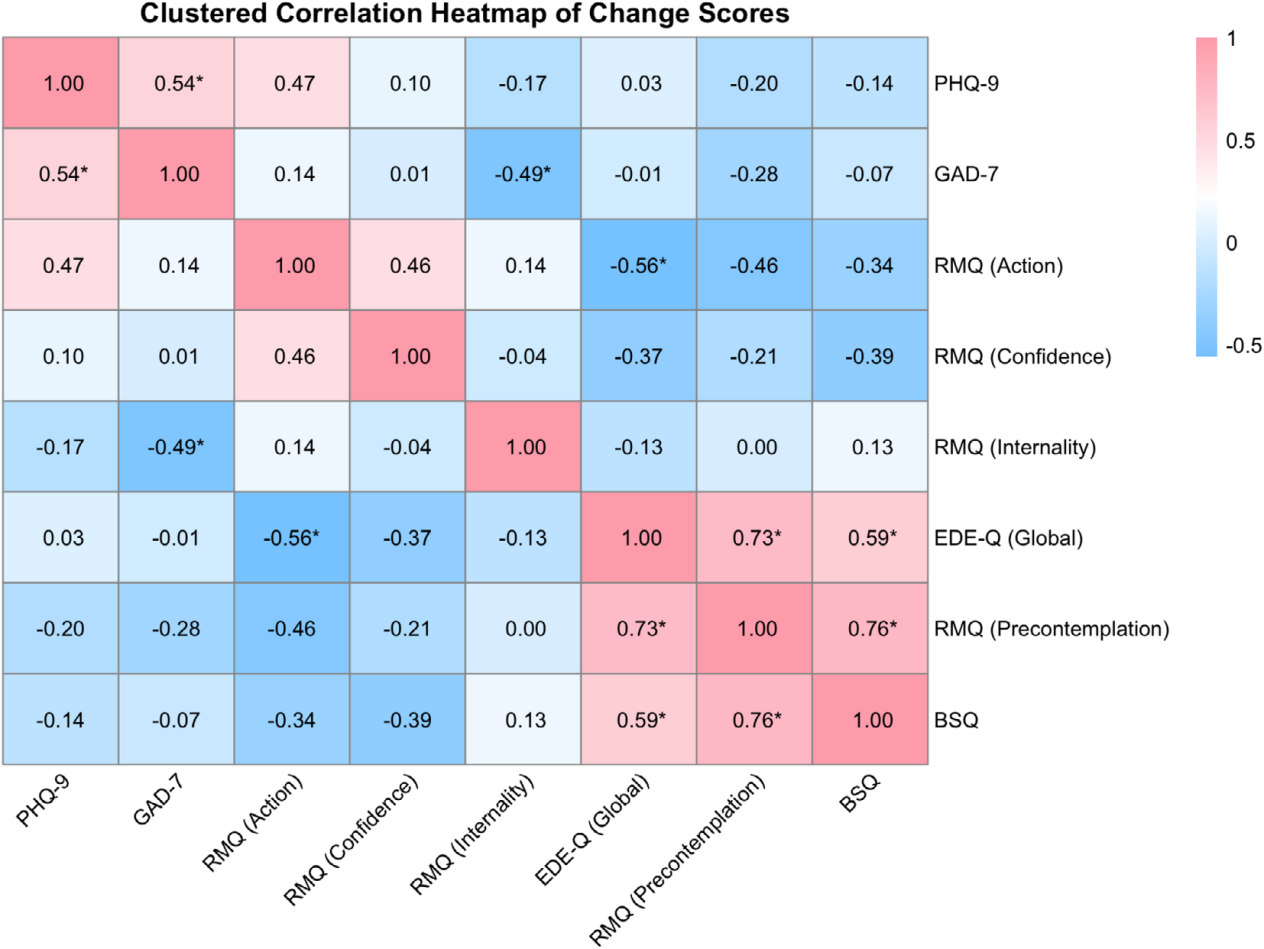
Clustered correlation heatmap of change scores. This figure displays Pearson correlations among change scores from pre‐ to post‐intervention across all motivational and clinical outcome measures. Positive associations are shown in red; negative associations are shown in blue. RMQ = readiness and motivation Questionnaire; EDE‐Q = eating disorder examination questionnaire; PHQ‐9 = patient health questionnaire–9; GAD‐7 = generalized anxiety disorder–7; BSQ = body shape questionnaire. *p < 0.05.

To better understand how changes in motivation and symptoms co‐occurred over the course of the intervention, Pearson correlations were calculated among change scores across all key clinical and motivational measures (see Figure 3). Several moderate associations were observed across select measures. Changes in ED symptoms, as measured by the EDE‐Q Global score, were moderately to strongly correlated with reductions in RMQ Precontemplation scores. EDE‐Q global scores were also moderately correlated with changes in body dissatisfaction, as measured by the BSQ. Changes in RMQ Precontemplation scores were strongly associated with changes on the BSQ. Change scores on the PHQ‐9 and GAD‐7 were also moderately correlated. In contrast, a moderate positive correlation was observed between RMQ Confidence and Action scores. These correlations describe patterns of co‐occurrence across motivational and symptom change scores and are reported for exploratory, descriptive purposes only.

## 4 Discussion

The current study evaluated the feasibility, acceptability, and preliminary outcomes of *MI‐Coach: ED*, a mobile app‐delivered, program‐led intervention developed for individuals on ED treatment waitlists. By integrating evidence‐based MI strategies into a scalable, self‐guided digital platform, the intervention aimed to provide accessible support during a period of elevated risk and limited access to care.

### 4.1 Feasibility and Recruitment

Study findings point to both the potential and complexity of delivering app‐based support during the waitlist period. Feasibility was substantially constrained at the service‐provider level: only 6% of contacted treatment sites agreed to distribute study materials to individuals on their waitlists, indicating limited provider uptake. Many sites lacked formal waitlist infrastructure or ongoing contact with waitlisted individuals, precluding estimation of underlying waitlist size or recruitment yield relative to a known denominator. Low provider participation represents a significant feasibility barrier, as sustained implementation of waitlist interventions depends on alignment with service workflows, administrative capacity, and perceived clinical value (Graham et al. 2023). In contrast to these provider‐level constraints, feasibility at the participant level was primarily reflected in patterns of app engagement and retention.

### 4.2 Engagement Patterns

At the participant level, engagement with *MI‐Coach: ED* was variable. Of enrolled participants, 67.6% initiated app use, 44% completed at least four of seven modules, and one participant completed all modules, indicating partial program exposure for most participants. Overall attrition was 22%, with most participants completing post‐intervention assessments. These engagement rates are lower than those reported for digital interventions targeting depression and anxiety, yet consistent with ED‐specific digital interventions. In ED samples, median module completion rates of approximately 36% and post‐assessment attrition of 21%–25% have been reported (Linardon, Shatte, Messer, et al. 2020), whereas general mental health apps show higher uptake (92.4%) and lower attrition (18.6%; Liu et al. 2026). Together, these findings suggest that feasibility benchmarks derived from general mental health apps may overestimate typical engagement in ED populations. Importantly, engagement with digital interventions is increasingly conceptualized as extending beyond usage metrics alone to involve self‐regulated learning processes such as reflection, goal alignment, motivation, and self‐regulatory capacity (Liu et al. 2025). Accordingly, engagement findings are interpreted as indicators of uptake and sustained use, while highlighting the need for longer trials to examine how different forms of engagement relate to outcomes over time.

### 4.3 Acceptability and Perceived Usefulness

Participant feedback further indicated that *MI‐Coach: ED* was broadly acceptable among those who engaged with the app. Ratings of app quality, usability, and flexibility were high, and most participants indicated that they would recommend the app to others. These findings complement qualitative data showing that users and clinicians viewed the app as accessible and supportive during prolonged wait periods (Halicki‐Asakawa, Gerlof, et al. 2025). Perceived usefulness varied across participants, aligning with prior pilot studies suggesting that brief digital tools may benefit some users more than others (Cardi et al. 2013; Levin et al. 2017). Importantly, high acceptability co‐occurred with variable engagement, consistent with evidence that satisfaction does not guarantee sustained use in digital ED interventions (Cruz et al. 2025; Graham et al. 2023; Schlegl et al. 2020; Yim and Schmidt 2019).

### 4.4 Observed Clinical Patterns

Across the 4‐week period, most participants remained stable on motivational and symptom measures. Clinical significance classifications primarily reflected unchanged scores, with a small number of participants showing improvement or deterioration across symptom and motivational domains. Confidence intervals around mean change scores and standardized effect sizes spanned zero for most outcomes, indicating substantial uncertainty in the magnitude and direction of change. Descriptive correlations suggested co‐occurring changes across select measures, including associations between reductions in RMQ Precontemplation and changes in EDE‐Q Global and BSQ scores. Relative to earlier MI‐based ED pilot studies reporting greater motivational than symptom change (Feld et al. 2001; Weiss et al. 2013), greater variability was observed in symptom‐related outcomes, likely reflecting natural fluctuation during the waitlist period rather than intervention‐related effects. These findings, though descriptive in nature, highlight motivation as a relevant construct for future investigation in ED waitlist interventions.

### 4.5 Implications for Design and Implementation

These findings underscore the importance of implementation strategies that reduce administrative burden and align with existing service workflows. In response, the ongoing RCT incorporates pilot‐informed modifications aligned with human‐centered design principles (Graham et al. 2023), including formal partnerships with provincial health authorities, recruitment through established waitlists, asynchronous video‐based onboarding, removal of the initial telephone screener, and streamlined assessment batteries. These changes are intended to embed recruitment within routine workflows and reduce burden on both clinicians and participants.

From a user‐engagement perspective, these findings suggest several directions for improving appeal among ambivalent individuals with EDs. Consistent with human‐centered design principles, future iterations may benefit from increased flexibility in pacing, clearer upfront framing of module expectations, and greater emphasis on low‐effort entry points, such as brief or optional activities, to support engagement when motivation is low or variable (Graham et al. 2023; Linardon, Shatte, Messer, et al. 2020). Qualitative feedback from participants emphasized the importance of feeling emotionally safe and able to engage at a manageable pace, which further underscores the potential value of optional content pathways, brief reflective exercises, and normalization of partial or intermittent use rather than linear completion (Halicki‐Asakawa, Gerlof, et al. 2025). Together, these considerations emphasize the importance of designing waitlist interventions that accommodate ambivalence and fluctuating readiness for change, rather than relying on sustained or sequential engagement.

### 4.6 Limitations

Several limitations should be acknowledged. The sample lacked diversity, with most participants identifying as Caucasian and cisgender women. Recruitment through treatment centers may have underrepresented individuals facing upstream barriers to care, including racially marginalized or gender‐diverse individuals, reflecting broader systemic inequities in ED service access (Becker et al. 2003; Goel et al. 2023; Gordon et al. 2006; Rodgers et al. 2020). Findings may also not generalize to individuals with limited digital access or older adults. Although the app was designed to be inclusive, no participants identified as gender‐diverse, limiting conclusions about its appropriateness for these groups.

Limited information was available from participants who disengaged, restricting insight into reasons for dropout. Engagement and acceptability estimates reflect app initiators only, as participants who did not begin app use were lost to follow‐up and excluded from pre‐post analyses. The use of a completer sample rather than an intent‐to‐treat approach further limits the generalizability of feasibility and preliminary outcome estimates. As a feasibility‐focused pilot with a small sample, the study was not designed to assess efficacy. Nonetheless, observed descriptive patterns, alongside strong engagement and acceptability, support continued evaluation of *MI‐Coach: ED*, with pre‐specified feasibility benchmarks met. A larger RCT is underway and incorporates pilot‐informed refinements, including embedding recruitment within clinical workflows, streamlined onboarding, and targeted outreach to improve reach, representation, and real‐world feasibility.

## 5 Conclusion

*MI‐Coach: ED appears* acceptable at the participant level while highlighting substantial provider‐level barriers to implementation. By offering structured support during a neglected phase of care, *MI‐Coach: ED* shows promise as a complementary model to existing stepped‐care approaches. Future research should continue to address both system‐ and participant‐level barriers to uptake in larger trials.

## Author Contributions

**A.H.‐A.:** conceptualization, methodology, investigation, resources, data curation, formal analysis, writing – original draft preparation, writing – reviewing and editing, project administration. **E.F.:** data curation, formal analysis, writing – original draft preparation. **M.L.:** conceptualization, validation, resources, writing – reviewing and editing, supervision.

## Funding

A. Halicki‐Asakawa was supported by a Canada Graduate Scholarships–Master's award from the Canadian Institute for Health Research (CIHR).

## Disclosure

Involvement of persons with lived experiences. This study was informed by lived and professional expertise. One author (AHA) has lived experience of an eating disorder and recovery, which informed the conceptualization, design, and interpretation of the present study. The broader *MI‐Coach: ED* pilot program was also shaped through consultation with individuals with lived experience of eating disorders, clinicians, and community organizations during app development and the pilot phase.

## Conflicts of Interest

The authors declare no conflicts of interest.

## Supporting information


**Data S1:** eat70053‐sup‐0001‐supinfo.doc.

## Data Availability

The data that support the findings of this study are openly available in Open Science Framework at https://osf.io/4zn5b/, reference number 4zn5b.
